# Case Report: Two Cases of Abdominal Aggressive Fibromatosis That Mimicked Abdominal Wall Endometriosis and Review of Literature

**DOI:** 10.3389/fmed.2021.774235

**Published:** 2021-12-02

**Authors:** Xin Chen, Yuan Wang, Haiyuan Liu, Honghui Shi, Qingbo Fan, Jinghe Lang

**Affiliations:** Department of Obstetrics and Gynecology, National Clinical Research Center for Obstetric and Gynecologic Diseases, Peking Union Medical College Hospital, Chinese Academy of Medical Sciences, Peking Union Medical College, Beijing, China

**Keywords:** abdominal wall mass, aggressive fibromatosis, differential diagnosis, endometriosis, periodic symptoms

## Abstract

**Background:** Abdominal aggressive fibromatosis (AF) can be confounded with abdominal wall endomentriosis (AWE) because they share considerable similarity. Because of the different patient prognoses and treatment strategies available, accurate pre-operative diagnosis is important.

**Case Presentation:** We here report two cases of abdominal masses presenting as periodic changes in tumor sizes, which occurred in correlation with the menstrual cycle. The clinical findings were highly suggestive of AWE. However, the final pathological findings revealed AF. The estrogen receptor and progesterone receptor expressions were negative in the two cases. The differences between the two diseases have been discussed in detail.

**Conclusion:** A diagnosis of AWE should be scrutinized closely if the patient does not complain of cyclic pain. Fine-needle aspiration cytology is a suitable tool for pre-operative evaluation.

## Introduction

If a mass within or adjacent to a cesarean section scar is found in female patients of reproductive age, what is the first consideration? For many doctors, especially gynecologists, abdominal wall endometriosis (AWE) might be the first clinical diagnosis. However, other diseases, such as aggressive fibromatosis, can also occur in similar demographics and locations within the body. Aggressive fibromatosis (AF), also called desmoid-type fibromatosis (DF), is a benign monoclonal fibroblastic proliferation that arises in the deep soft tissues (1). AF is characterized by infiltrative growth and a tendency toward local recurrence but an inability to metastasize (1). AF can be further subdivided into extra-abdominal, abdominal, and intra-abdominal types (2). Both abdominal AF and AWE are inclined to occur in young women with a history of cesarean section and can appear as solitary masses with infiltrative margins, which can render pre-operative clinical diagnosis difficult (2–5). Although there are a few reports in the literature about misdiagnosis of AF as AWE, few of them have discussed the differences between the two diseases in detail. Here, we report two cases of abdominal AF that mimicked AWE and summarize differences between the two entities through literature review in order to guide proper pre-operative diagnosis and foster strong surgical outcome.

## Case Presentation

### Case One

**Figure 1:**
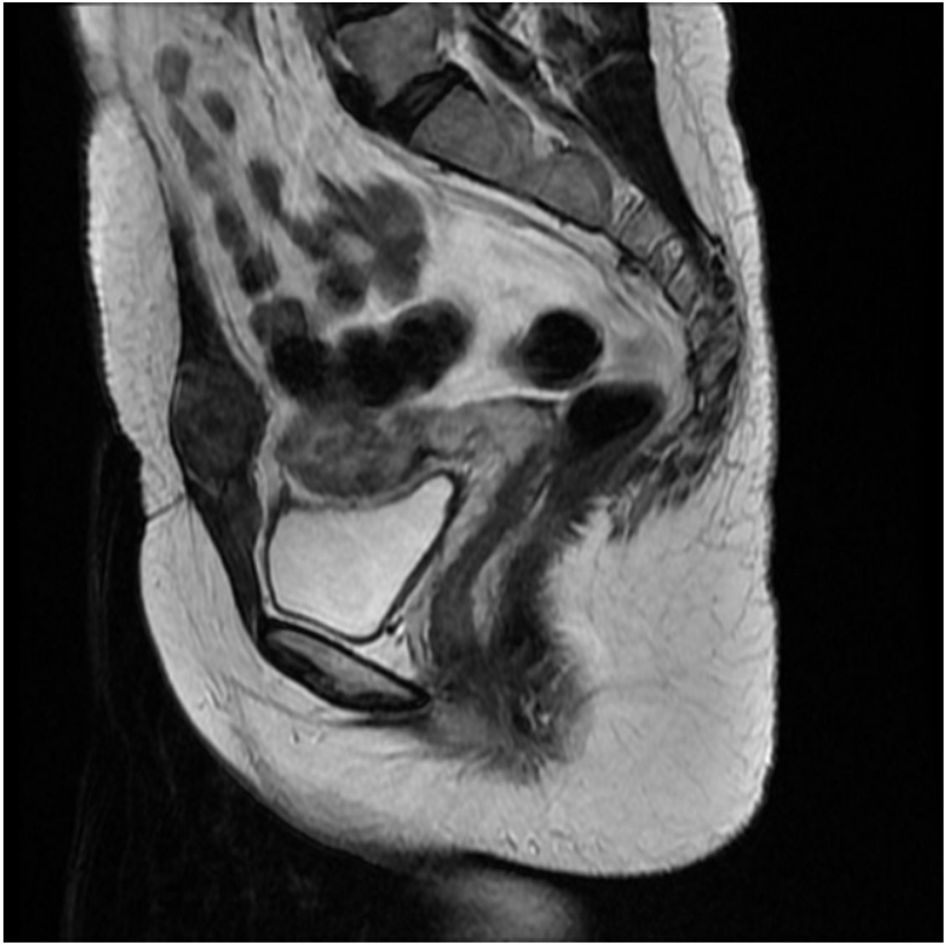
Magnetic resonance imaging (MRI) revealed a 3 × 2.6-cm soft tissue mass in the anterior abdominal wall.

A 35-year-old woman presented to our hospital with the complaint of a painless mass on the lower abdominal wall for 4 months. This gravida 1 para 1 patient had undergone a cesarean section operation 3 years earlier without any known post-operative complications. The mass was notably enlarged during the menstrual period. A 4-cm, round, solid, painless, fixed mass above the left side of cesarean section incision was detected upon physical examination. Ultrasound revealed a hypoechoic and avascular mass of 4.9 × 3.5 × 2.2-cm in diameter located in the subcutaneous muscle layer below the cesarean section incision. MRI revealed an abnormal signal of slightly hyperintense on T2WI and iso-intense on T1WI with enhancement, located in the left rectus abdominis with a maximum section of 3 × 2.6-cm (Figure 1).

With a pre-operative diagnosis of AWE, a wide surgical excision was then performed. The patient provided written informed consent. At surgery, a 4-cm mass was found in the extraperitoneal musculature, which was completely excised with a 1-cm tumor-free margin. Each layer opened was closed with suture and no mesh was needed. The tumor had no obvious capsule and when sectioned it was beige and firm and showed a swirling pattern. The pathological findings revealed AF. The post-operative course was uneventful. No recurrence occurred during the 2-year follow-up.

## Case Two

A 39-year-old woman presented with an abdominal wall mass for 5 months. This gravida 4 para 2 patient had undergone cesarean section operations 12 and 3 years earlier. The abdominal wall mass increased during menstruation and decreased after menstruation. Physical examination revealed a 6 × 5-cm, fixed and firm abdominal mass on the right side of the scar. Ultrasound showed a 6.0 × 3.2 × 1.7-cm hypoechoic irregular mass in the muscular layer with a fuzzy boundary and hemogeneous internal echo. Color Doppler ultrasonography showed several strip blood flow signals inside with the peak systolic velocity (PSV) of 15.5 cm/s and resistance index (RI) of 0.77.

The clinical findings suggested the mass might be AWE. A wide local excision of the mass was performed. The patient provided written informed consent. At surgery, the lesion was found in the rectus abdominis under the fascia. The lesion was resected along the outer edge of the lesion and did not enter the abdominal cavity. The cut section showed areas of beige color. It was firm and solid. Histopathology confirmed desmoid-type fibromatosis. All margins were negative. Immunohistochemical findings were positive for SMA, β-catenin and CD34 (vessel), whereas desmin, Caldesmon and S-100 were negative. Fewer than 3% of cells were Ki67-positive. The patient is on follow-up with no clinical signs of recurrence after 4 years.

## Discussion

Aggressive fibromatosis (AF) is a distinct rare entity with an incidence of five to six cases per 1 million of the population per annum and a peak age of 30–40 years (2). The exact etiology is not fully understood. Documented etiological factors are surgical trauma, genetic factors (e.g., familial adenomatous polyposis, FAP), hormonal influences, and pregnancy (1, 2). Abdominal AF can occur in any part of the abdominal wall, mainly in the lower abdomen. It usually presents as a painless, solitary, and fixed mass in the deep layer (2). Abdominal AF usually involves muscle or aponeurosis and presents as a single lesion but rarely can be multifocal (6). At MRI, the lesions appear as soft-tissue masses with heterogeneous internal signal on all sequences with avid enhancement, reflecting their proportionate cellular and fibrous contents (2, 6, 7). One hallmark feature is the presence of linear or sheet intra-lesional hypointense bands on T2-weighted images (6, 8). The fascial tail, described as the linear extension of the tumor along the fascial planes, is also a highly suggestive feature (7, 8). Macroscopically, the section of AF mass is beige, swirling, and firm, and it often infiltrates the adjacent muscles and aponeurosis (1). Histologically, AF is characterized by a fibromatous, benign proliferation of well-differentiated fibroblast and tentacle-like spiculated extensions with infiltrative growth (1). Approximately 85–90% of AF has nuclear positivity for ß-catenin, which is helpful in establishing the diagnosis (1).

Surgery is no longer the first-line treatment of AF because of the variable and unpredictable clinical course. Currently, a conservative wait-and-see policy for 1–2 years is the front-line approach to newly diagnosed patients, irrespective of clinical symptoms (1). In cases that progress, anti-hormonal therapy or surgical resection might be an option (1). Pharmacological options include anti-hormonal therapies such as tamoxifen, non-steroidal anti-inflammatory drugs (NSAIDs), and low-dose chemotherapy (1). It is necessary to remove the tumor with a margin of at least 2–3 cm to make sure a negative margin (9).

The clinical course of AF is variable and often unpredictable. Spontaneous regressions are observed in 20–30% of cases (1). The risk of progression during pregnancy is as high as 40–50% (1). The rate of local recurrence rate of abdominal AF after surgery ranges from <10 to 40% (1, 2).

The differences in clinical characteristics are summarized in Table 1. The two aforementioned cases were suspected for AWE because changes in size that correlate with the menstruation cycle are highly suggestive. However, the two patients did not complain of cyclic pain. A diagnosis of AWE should be scrutinized closely if the patient does not complain of cyclic pain. To our knowledge, this is the first report of periodic changes in size of abdominal AF.

**Table 1:** Differences of clinical features between abdominal AF and AWE.

**Items**		**Abdominal AF**	**AWE**
Incidence		0.0005%	0.04–12% (10)
Etiology		Trauma, hormones, and genetic factors	Iatrogenic implantation of endometrium (5)
Typical symptoms		A painless and fixed mass	Cyclic abdominal pain (3, 5, 10)
Involved layer		Muscle or aponeurosis	Adipose (8)
MRI		Linear and sheet hypointense bands on T2W The fascial tail	High T1 signal intense that does not diminish with fat saturation signal (8)
Pregnancy		40–50% of progression	No progression
Pathology	Section appearance	Beige, swirling and firm	Micro-cysts with a chocolate-like appearance (4)
	Microscopic appearance	Well-differentiated fibroblast	Endometrial glands, stroma, and hemosiderin (5)
Treatment	Primary option	A wait-and-see policy	Surgical resection (5)
	Pharmacological treatment	Effective	Less effective (10)
	Resection range	At least 2–3 cm	1 cm (5)
Prognosis	Malignance	No metastasis	1% (3, 5)
	Recurrence	10–40%	4.3–5.9% (4, 10)

*AF, Aggressive fibromatosis; AWE, Abdominal wall endometriosis*.

In addition to cyclic pain, there are other clinical features for differentiation. The more common position for AWE is the adipose layer, while abdominal AF usually involves muscle or aponeurosis (6, 8). On MRI, the appearances of AF depend on the proportion of cellular and fibrous contents whereas those of AWE depend on lesion chronicity (2, 6, 7). The most common diagnosis of abdominal wall lesions exhibiting a high T1 signal that does not decrease with fat saturation signal is endometriosis (8). The section of the AWE mass is usually yellowish with areas of hemorrhage or chocolate-like micro-cysts (4), unlike AF. Thus, if the typical section is not found upon the removal of AWE, other diseases should be considered. A pathological examination of frozen samples should be made to make sure the proper resection range is used. Occurrence or progression during pregnancy or coexisting FAP are also suggestive of AF.

**Figure 2:**
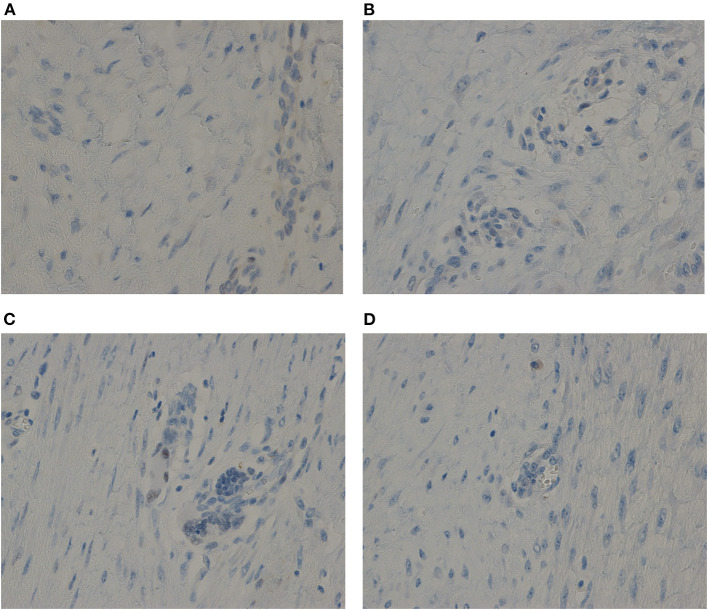
The immunohistochemical testing showed negative results for both ER and PR in the two cases. **(A)** For ER of Case one. **(B)** For PR of Case one. **(C)** For ER of Case two. **(D)** For PR of Case two. Anti-ER or Anti-PR antibody immunostaining, × 40.

AF oncogenesis is associated with estrogen hormonal stimulus (2). To determine the causes of the periodic symptoms in our cases, the levels of expressions of estrogen receptors (ERs) and progesterone receptors (PRs) were here verified. The immunohistochemical testing showed negative results for both ER and PR in the two cases (Figure 2).

Several differential diagnoses need to be considered, such as disseminated peritoneal leiomyomatosis (DPL), parasitic leiomyoma, abdominal wall metastase and soft tissue sarcomas. DPL is defined as the presence of multiple peritoneal/sub-peritoneal nodules of various sizes composed of bland smooth muscle cells. In one of the largest cohorts of DPL, 29% of DPL and 60% of malignant DPL had abdominal wall involvement (11). Parasitic leiomyoma is a rare complication of power morcellation following laparoscopic myomectomy or hysterectomy, in which the fragment of myoma may be trapped somewhere along the trocar tract in the abdominal wall (12). Abdominal wall metastasis comprise tumors that reach the abdominal wall by implantation, direct invasion and metastasis. Imaging appearances are usually non-specific, often resembling other sites of primary disease (8). Implantation cancers have been reported to occur in 1.18% of patients with gynecological malignancies after laparoscopy (13). Soft tissue sarcomas usually occur later in life, with a median age at presentation of ~50 years. Fixation to underlying structures is suggestive of a soft-tissue sarcoma (14). Therefore, patients with abdominal wall masses should be referred to specialist centers benefiting from multidisciplinary teams experienced in the management of soft tissue tumors.

## Conclusion

AF and AWE are important conditions that should be considered in the differential diagnosis of masses located at cesarean section scars. Cyclic pain is an important differential point. Imaging is a helpful tool for diagnosis but its value is limited. In cases without typical manifestation, fine-needle aspiration cytology can help to prevent mistakes. Furthermore, a multidisciplinary team should be recommended.

## Data Availability Statement

The original contributions presented in the study are included in the article/supplementary material, further inquiries can be directed to the corresponding author.

## Ethics Statement

Written informed consent was obtained from the individual(s) for the publication of any potentially identifiable images or data included in this article.

## Author Contributions

HS, HL, QF, and JL diagnosed the patients. YW did the immunohistochemical test. XC wrote the manuscript. All authors revised the manuscript.

## Conflict of Interest

The authors declare that the research was conducted in the absence of any commercial or financial relationships that could be construed as a potential conflict of interest.

## Publisher's Note

All claims expressed in this article are solely those of the authors and do not necessarily represent those of their affiliated organizations, or those of the publisher, the editors and the reviewers. Any product that may be evaluated in this article, or claim that may be made by its manufacturer, is not guaranteed or endorsed by the publisher.
